# The Accuracy of Sex Identification Using CBCT Morphometric Measurements of the Mandible, with Different Machine-Learning Algorithms—A Retrospective Study

**DOI:** 10.3390/diagnostics13142342

**Published:** 2023-07-11

**Authors:** Mohammed Taha Ahmed Baban, Dena Nadhim Mohammad

**Affiliations:** 1 Department of Dental Nursing, Sulaimani Technical Institute, Sulaimani Polytechnic University, Sulaimani 46001, Iraq; 2 Department of Oral Diagnosis, College of Dentistry, University of Sulaimani, Sulaimani 46001, Iraq

**Keywords:** forensics, sex determination, CBCT, mandible, machine learning

## Abstract

In forensics, predicting the sex is a crucial step in identification. Many studies have aimed to find an accurate and fast technique to estimate the sex. This study was conducted to determine the accuracy of volumetric and linear measurements of three-dimensional (3D) images of the mandible obtained from cone beam computed tomography (CBCT) radiographs, using different machine-learning (ML) models for sex identification. The CBCTs of 104 males and 104 females were included in this study. The radiographs were converted to 3D images, and the volume, surface area, and ten linear measurements of the mandible were obtained. The data were evaluated using statistical analysis and five different ML algorithms. All results were considered statistically significant at *p* < 0.05, and the precision, recall, f1-score, training accuracy, and testing accuracy were used to evaluate the performance of the ML models. All the studied parameters showed statistically significant differences between sexes *p* < 0.05. The right coronoid-to-gonion linear distance had the highest discriminative power of all the parameters. Meanwhile, Gaussian Naive Bayes (GNB) showed the best performance among all the ML models. The results of this study revealed promising outcomes; the sex can be easily determined, with high accuracy (90%).

## 1. Introduction

Human identification is one of the most important aspects of forensic odontology. The identification of deceased persons is essential for social and legal closure [1]. Forensic dentistry is critical in recognizing individuals’ unique dental and facial features. It is regarded as a powerful tool for the identification of human remains in events where highly damaged and dismembered dead bodies are recovered from mass graves, earthquakes, tsunamis, airplane crashes, train and road accidents, bomb blasts, and wars [2].

Sex determination is considered an essential step in forensic and anthropological human identification. It narrows the search for the deceased person’s identity, by excluding approximately one-half of the population [3]. Skeletal evaluation is a very accurate and reliable method for sex determination; in particular, the accuracy of morphometric measurements of the pelvis can reach up to 95% [4]. However, in some instances, sex determination can be very challenging for forensic experts, when only a few fragments of a human skeleton have been recovered [5].

The mandible is a probable finding by forensic experts, as it is the strongest bone in the human face, characterized by its compact nature and higher resistance to degradation by environmental factors [6]. To overcome morphological sex estimation subjectivity and experience-related error, many studies have been conducted to evaluate the mandible morphometrically, to identify its ability to predict sex accurately. Interestingly the literature shows that the mandible’s morphometric parameters have significant sexual dimorphism, and can determine the sex accurately, making it the best choice for sex estimation, after the pelvis [7,8,9].

In forensics, various imaging techniques have been used to evaluate the morphometry of human skeletal remains. Surface scanning is commonly used, to provide a 3D topographic dataset for skeletal remains. The widely used 3D surface scanners are structured-light systems and laser scanners. However, surface-scanning techniques have many disadvantages, such as the fact that the scanning process can create noise, especially when scanning teeth because light reflection by the enamel is higher than the normal intensity. Another limitation of surface scanners is the difficulty in scanning and collecting the internal morphometry and density of bones with an irregular morphology (such as the bones of the maxillofacial region) [10]. Another method of obtaining a 3D image is using coordinate-measuring machines. Nevertheless, structures that have a complex morphology, such as the bones of the cranium, are very time-consuming to scan, and sometimes it is impossible to create 3D images of these structures using coordinate-measuring machines [11].

Radiographical investigations play an essential role in the sex identification of the decomposed body [1]. Linear morphometric measurements are obtained from two-dimensional radiographs. In contrast, 3D image radiographs with analysis software provide accurate linear and volumetric measurements of different structures in the human skull [12]. Furthermore, the newly invented CBCT with high resolution provides a precise segmentation that enables the operator to obtain data for individual structures, and overcome overlapping structures, as in two-dimensional radiographs [13]. Interestingly, studies conducted using CBCT radiographs outperform research using more traditional radiographs, in the diagnostic examination of deceased persons [14].

ML is the art and science of getting computers to learn and act as humans do, and to autonomously improve their learning over time, by feeding them data to make predictions [15]. ML can be used in forensic odontology to identify individuals through their remains. Studies have shown that ML can be used for age estimation, sex determination, bite-mark analysis, and dental identification, with promising results [16,17,18].

Supervised learning is a subtype of ML that uses labeled examples to teach the algorithm how to predict output variables, from input variables. The supervised ML could be applied in various applications, such as manufacturing, marketing, finance, the stock market, testing, prediction, and many medical fields [19].

A decision tree (DT) is a type of supervised ML that recursively divides the data into subsets, according to the values of the independent variables. It creates a model that predicts the value of a target variable, based on several input variables [20]. The random forest (RF) algorithm generates several decision trees, from randomly chosen subsets of data. Each tree is trained on a separate subset of the data, making a prediction. The final prediction is then created by merging all the trees’ predictions [21].

The k-nearest neighbors (KNN) algorithm is an ML algorithm used in classification and regression. It operates by locating the k-nearest neighbors to a given data point, and using their labels to make predictions. The value of k controls how many neighbors are taken into account [22]. The GNB algorithm is based on applying Bayes’ theorem, a probabilistic classification model with strong independence assumptions [23]. In the context of classification, independence refers to the idea that the presence of one value of a feature does not influence the presence of another. Logistic regression (LR) is a classification algorithm used to solve classification problems. It determines the best-fit line to describe the relationship between the independent and dependent variables, and then the line is used to make predictions about new data [24].

Despite the intensive use of ML models in forensic research, limited studies have been conducted using ML algorithms to estimate sex from 3D radiographs of the mandible. This study aims to evaluate the accuracy of morphometric measurements of the mandible, using different ML algorithms, to predict the sex. The null hypothesis is that it is impossible to predict the sex with high accuracy by using ML models to analyze morphometric measurements of CBCT radiographs of the mandible.

## 2. Materials and Methods

### 2.1. Study Design

This study was conducted from March 2022 to February 2023. The ethical approval of this study was obtained from the Ethics Committee at the College of Dentistry/University of Sulaimani-No. 36/21, on 11 August 2021. This research was performed following the relevant guidelines. The authors waived informed consent, as they analyzed archives of CBCT radiographs.

A total of 687 CBCT radiographs had been scanned from the archive of Foton Maxillofacial Imaging Clinic in Sulaimani/Iraq. Radiographs that showed the entire mandible and belonged to subjects aged 18 years and above from both sexes were included in this study. On the other hand, CBCTs that met the following criteria were excluded:

1. CBCTs did not show the entire mandible, or showed less than ten remaining teeth.
2. Certain pathological conditions affected the mandible’s size and shape.
3. CBCT images contained metal in or near the mandible, or radiographs showed bone resorption around the lower teeth that reached the apical third of the roots.

As a result of the inclusion and exclusion criteria, 479 CBCTs were excluded, and only 208 radiographs were included in this study (104 females and 104 males), aged 18–70 years.

All the CBCT images were acquired using the CS 9600 unit manufactured by Carestream Dental, Atlanta, GA, USA., with the following technical specifications: 15.4 cm spherical imaging volume, 150 µm × 150 µm × 150 µm voxel size, and a field of view of 16 × 12 cm. All radiographs were taken according to the following parameters: 120 kVp, 5 mA, and an exposure time of 40 s.

### 2.2. Image Analysis

The CBCT radiographs were exported to multiple digital imaging and communications in medicine (DICOM) file formats, using CS 3D Imaging version 3.10.21, developed by Carestream Dental, Atlanta, GA, USA. The DICOM files were imported into RealGIUDE version 5.0 software, developed by RealGUIDE™ Software Suite Milan, Italy. The software automatically creates a 3D image of the mandible, facial bones, and other bones included in the radiograph.

All bones were eliminated manually, by using the sculpting tool of the software, except for the mandible. The following settings were used in the bone segmentation section of the software: the bone threshold was set to 400, the background was set to 300, the segmentation quality was set to 80%, the smooth was set to 2, and the maximum number of objects was set to 1 (to eliminate other objects, and keep the largest object, which was the mandible). Using the segment tool, the software automatically detected the boundaries of the mandible, then the mandible was segmented and the mesh was created. By using the remove, add, smooth, and fill tools of the software, any minor defects were corrected manually. The 3D images were exported in the stereolithography (STL) file format, using the Export Anatomy tool in the Report/Export section of the software.

**Figure 1:**
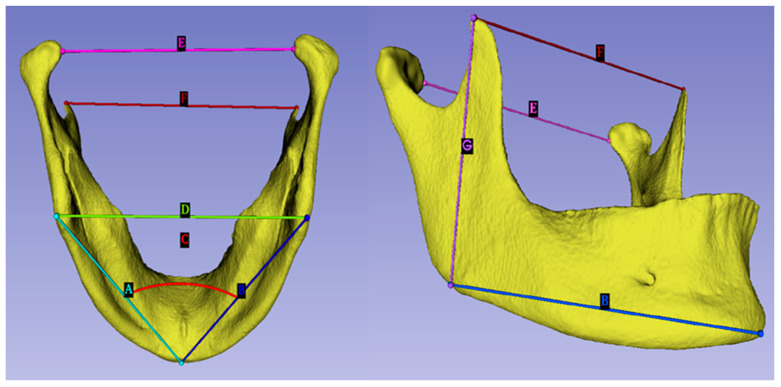
The linear measurements of the mandible: (A) left gonion to menton linear distance (LGonMenLD), (B) right gonion to menton linear distance (RGonMenLD), (C) right gonion to menton left gonion angle (RGonMenLGonA), (D) bigonial breadth (BigonB), (E) bicondylar breadth (BiconB), (F) bicoronoid breadth (BicorB), (G) right coronoid to gonion linear distance (RCorGonLD).

The Vol and the SA of the mandible were obtained, using the Meshmixer version 3.5.474 software, developed by Autodesk corporate, San Francisco, CA, USA. The STL files were opened in the 3D Slicer version 4.13.0 software, an open-source software platform for biomedical research, and all linear measurements (as shown in Figure 1) were taken manually by a single examiner. The intra-examiner reliability test was conducted on 30 randomly collected samples, after six months. The surface area/volume (SA/Vol) ratio, the mean coronoid-to-gonion linear distance (mean CorGonLD), and the mean gonion-to-menton linear distance (mean GonMenLD) were calculated using Microsoft Excel 2021, developed by Microsoft Corporation, Redmond, WA, USA.

### 2.3. Statistical Analysis

The data were analyzed using Statistical Package for The Social Sciences (SPSS) Version 25, developed by International Business Machines Corporation (IBM), New York, NY, USA. The intraclass correlation coefficient was used to assess the intra-examiner reliability, and descriptive statistics were used to calculate the mean ± standard deviation (SD), median, and interquartile range (IQR).

The normality of the data was tested using the Shapiro–Wilk normality test. The nonparametric data were analyzed using the Mann–Whitney U test, while the independent *t*-test was applied to the parametric data. To reveal the discriminative power of each morphometric measurement of the mandible when predicting the sex, an ROC (receiver operating characteristic) analysis was applied. All results were considered statistically significant at less than *p* < 0.05.

### 2.4. Machine-Learning Algorithms

Anaconda Navigator version 2.1.4, and Jupyter Notebook version 6.4.8., developed by Anaconda Inc., Austin, TX, USA, in the Python programming language version 3.10 developed by The Python Software Foundation, Wilmington, DE, USA. were used in this study to create ML models. The ML modeling was carried out using 9th gen i7, MSI personal computer Model: GF65. The GNB, LR, DT, RF, and KNN algorithms were used to analyze the data. The dataset was split, with 80% designated as the training set, while the remaining 20% of the data was assigned as the test set. Furthermore, to assess the reliability of ML modeling, tenfold cross-validation of the accuracy values has been performed, by mixing the dataset through shuffling and running the ML algorithms ten times. The mean and SD of each ML algorithm’s accuracy were calculated using Microsoft Excel 2021, developed by Microsoft Corporation, Redmond, WA, USA.

#### Performance Criteria

The Sensitivity, Specificity, Accuracy, Recall, Precision, and F1 score values were included as performance criteria.

$$\mathrm{Sensitivity}=\frac{\mathrm{TP}}{\mathrm{TP}+\mathrm{FN}},\;\mathrm{Specificity}=\frac{\mathrm{TN}}{\mathrm{TN}+\mathrm{FP}},\;\mathrm{Accuracy}=\frac{\mathrm{TP}}{\mathrm{TP}+\mathrm{FN}+\mathrm{FP}+\mathrm{TN}}$$

$$\mathrm{Recall}=\frac{\mathrm{TP}}{\mathrm{TP}+\mathrm{FN}},\;\mathrm{Precision}=\frac{\mathrm{TP}}{\mathrm{TP}+\mathrm{FP}},\;F1\;\mathrm{score}=2\;\times \;\;\frac{\mathrm{Precision}\;\times \;\mathrm{Recall}}{\mathrm{Precision}\;+\;\mathrm{Recall}}$$

TP, true positive; TN, true negative; FP, false positive; FN, false negative.

**Figure 2:**
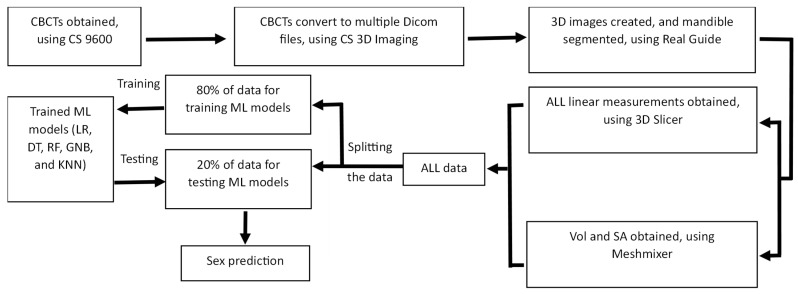
The flowchart diagram illustrates the process of image analysis and evaluation of data using ML models.

The process of taking the CBCTs, image analysis, and data-handling using ML algorithms is presented in Figure 2.

## 3. Results

**Figure 3:**
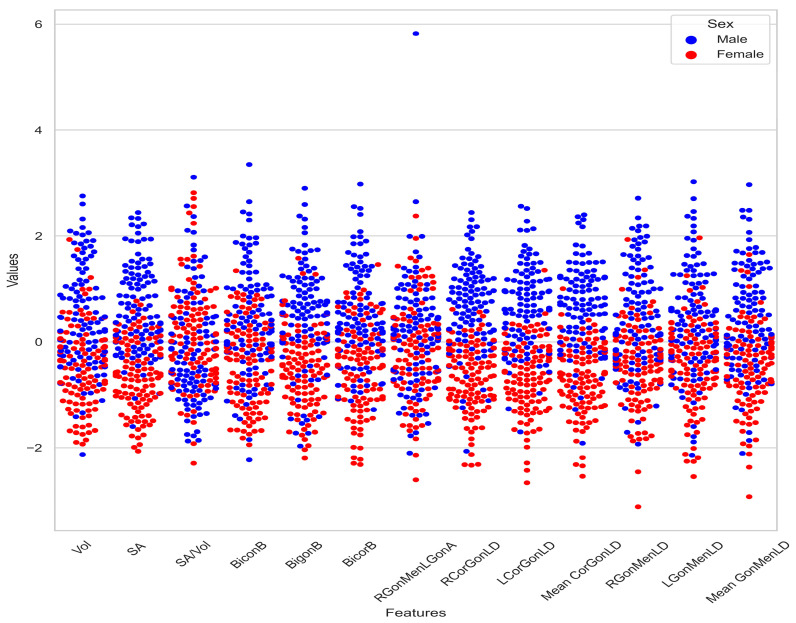
Scatterplot showing the morphometric values of the mandible in both sexes.

A total of 208 CBCTs were included in this study (104 males and 104 females). The mean ± SD age of the male subjects was 36.74 ± 13.71, while the mean ± SD age of the female subjects was 37.56 ± 14.58. The mean age between the male and female samples showed no significant difference (*p* = 0.678). The distribution of the morphometric values of the mandible in response to the sex is shown in Figure 3.

The normality test revealed that the BiconB, BigonB, BicorB, RCorGonLD, left coronoid-to-gonion linear distance (LCorGonLD), mean CorGonLD, LGonMenLD, and mean GonMenLD were normally distributed, while the volume (Vol), surface area (SA), SA/Vol, RGonMenLGonA, and RGonMenLD were nonparametric. The statistical relations of all parameters showed significant differences between males and females, with higher values in male subjects (Table 1 and Table 2).

**Figure 4:**
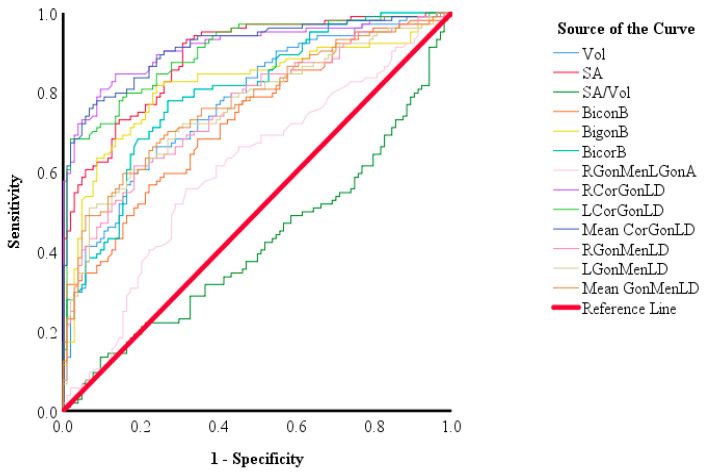
The ROC of all parameters.

An ROC analysis was conducted, to assess the discriminative power of each parameter in predicting sex. The ROC curves of all the mandible morphometric measurements are shown in Figure 4. The RCorGonLD and mean CorGonLD showed the higher AUC (0.914 and 0.913, respectively), followed by the LCorGonLD (0.901) and SA (0.888), while the SA/Vol showed the minimum discriminative power in estimating sex (AUC = 0.419) among all the morphometric mandible parameters (see Table 3). The ROC analysis showed statistical differences between all the parameters in males and females, with *p* < 0.05.

The RCorGonLD showed the highest sensitivity and specificity among all measurements (0.846 and 0.865, respectively), while the SA/Vol showed the lowest sensitivity and specificity (0.442 and 0.452, respectively). The AUC, cut-off value, *p* value, sensitivity, and specificity of all parameters are presented in Table 3.

**Figure 5:**
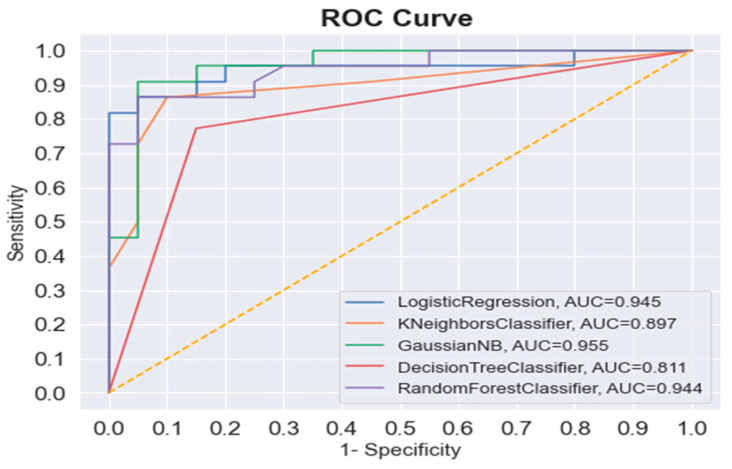
The ROC and AUC of all the ML algorithms (LR, KNN, GNB, DT, and RF) in sex determination (the dash line represents the reference line).

GNB demonstrated the higher discriminative power of all the ML algorithms (AUC = 0.955), followed by the LR and RF (0.945 and 0.944, respectively), while the AUC of the KNN classifier was 0.897. The ROC analysis of the DT algorithm showed the lowest AUC among all the ML models (AUC = 0.811). Figure 5 shows the ROC curve and AUC of all the ML algorithms.

**Figure 6:**
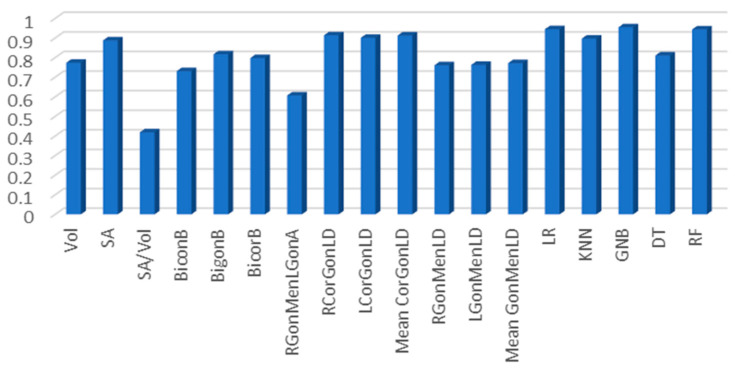
The AUC of the morphometric measurements of the mandible, and the ML algorithms.

Figure 6 shows the comparison between the discriminative power of all the anatomical measurements of the mandible and the ML models.

It was observed that GNB had the highest testing accuracy (0.90) and the lowest training accuracy (0.84) among all the ML algorithms, and the results of the confusion matrix of GNB predicted 19 of 20 females, and 19 of 22 males, correctly. This was followed by RF and LR (testing accuracy = 0.88), and the testing accuracy of the KNN model was 0.83. Meanwhile, the DT had the lowest testing accuracy (0.80) and highest training accuracy (1), with 17 of 20 females, and 17 of 22 males, correctly predicted, which were found in the confusion matrix of the DT. Regarding the f1-score, the GNB algorithm had the highest f1-score for males and females (0.90), succeeded by RF and LR (0.88). However, the DT demonstrated the lowest f1- score for both sexes (0.81). The precision, recall, and f1-score, as well as the training accuracy and the testing accuracy of all the ML algorithms, are presented in Table 4.

**Figure 7:**
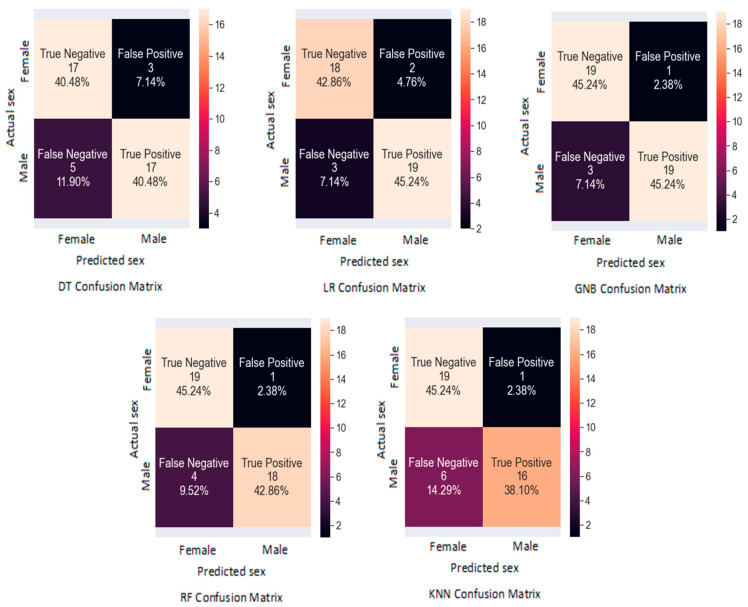
The confusion matrices of all the ML models (DT, LR, GNB, RF, and KNN).

The results of the confusion matrices of all the ML models are shown in Figure 7.

**Figure 8:**
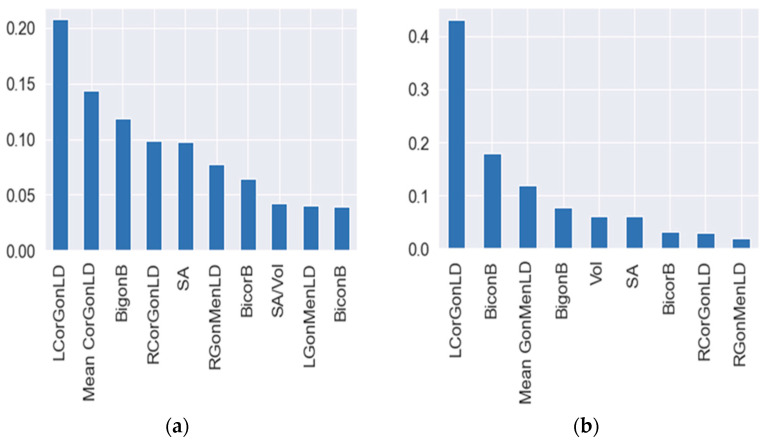
The mean (SHAP value) of the tested parameters, in response to: (**a**) RF, (**b**) DT.

The mean impact of each parameter on the RF and DT algorithms’ magnitude SHAP values has been used, as shown in Figure 8.

Regarding the reliability of this study, the result of the intraclass correlation coefficient, which was applied to assess the intra-examiner reliability of all parameters, ranged from 0.95 to 0.99. In addition, tenfold cross-validation was used to appraise the performance of the ML algorithms. The highest accuracy was obtained from KNN (0.848 ± 0.059) and LR (0.848 ± 0.068), followed by RF (0.84 ± 0.048), and GNB (0.84 ± 0.049), while DT showed the lowest accuracy (0.814 ± 0.042). The results of the tenfold cross-validation tests of all the ML algorithms are shown in Table 5.

## 4. Discussion

In forensic dentistry, various methods have been used to determine the sex of unknown human remains [25]. The radiograph is one of the most valuable tools in forensic odontology, because it gives objective evidence of the dental treatments, as well as the anatomical conditions, of the deceased person. In addition, it is a non-destructive, easy-to-use, and quick technique, which makes it cost-effective in comparison to molecular technology [26].

Medical image segmentation entails dividing DICOM images into distinct and meaningful segments. The selection of an appropriate threshold level is crucial for the segmentation of various structures in the skull. The 3D models of these structures are created by surface reconstruction, based on contour interpolation from different segments. The segmentation of 3D radiographic images is widely used in reconstructing anatomical structures of the cranium. The accuracy of 3D radiographic image reconstruction is influenced by the segmentation threshold range [27]. In this study, the mandible was segmented with minimal artifacts, by setting the bone threshold value to 400, and the background threshold value to 300.

Morphometric analysis of various bones of the human body has been used to predict the sex. The pelvis and cranium show the highest level of sexual dimorphism in the human skeleton. According to the literature, the possibility of finding an intact mandible is high, because the mandible is more durable than other bones in the cranium, and is composed of compact bone [28,29]. This study aimed to evaluate the performance of morphometric measurements obtained from CBCTs of the mandibles, using ML algorithms, in predicting the sex accurately.

In this study, all the morphometric measurements of the mandible (BiconB, BicorB, BigonB, LCorGonLD, LGonMenLD, mean CorGonLD, mean GonMenLD, RCorGonLD, RGonMenLD, RGonMenLGonA, SA, SA/Vol, and Vol) showed statistically significant differences between males and females *p* < 0.05. Males had higher mean values for all measured parameters than females. This can be attributed to the fact that during male growth, testosterone levels; and the more extended puberty phase, with a related longer duration of bone growth; affected the bone size. Another consideration is the muscular tension that encourages bone growth; because males have stronger masticatory muscles than females, the mandible is generally more developed in males [30,31].

The ROC findings of all the parameters revealed that RCorGonLD had the highest sensitivity and specificity in predicting the sex (AUC = 0.914). The most accurate ML model in estimating the sex was GNB (90%), followed by LR and RF, while DT showed the lowest prediction accuracy (80%). In this study, the AUC and ROC curve were used to measure the classifier’s ability to distinguish between males and females, the diagnostic efficiency of each morphometric mandible measurement in the prediction of the sex, and the cut-off values for each predictor. Researchers have indicated that using the ROC curve and AUC has advantages, such as the fact that the AUC is based on both specificity and sensitivity, and is unaffected by the prevalence of one investigated group over the other, in contrast to the single measures of specificity, sensitivity, and diagnostic accuracy. Furthermore, AUC and ROC curves can be used to compare different models, and are insensitive to class imbalance [32].

For this study, the null hypothesis was rejected, as sex could be determined with accuracy reaching up to 90%, by analyzing the morphometric measurements of the mandible using ML algorithms. The accuracy of sex estimation in this study appeared to be high, as the accuracy of sex prediction in previous studies that only evaluated the morphometric measurements taken from the mandible ranged between 53% and 90% [33,34,35,36,37,38,39,40].

A study conducted by Saloni et al. [33] showed that the sex could be predicted from the mandibular ramus, with accuracy reaching up to 77.6%, while Mehta et al. [34] reported that the sex could be estimated with the high accuracy of 77.3% from a minimum ramus breadth. Finally, Samatha et al. estimated the sex with an accuracy rate of 53% for males, and 60% for females. Still, a comparison with these results is difficult, as they used liner measurements from orthopantomogram radiographs (OPG) of the mandible, and analyzed them using basic statistics. In this study, CBCT radiography was used, which is considered the gold standard in oral and maxillofacial region imaging. Furthermore, unlike the 2D imaging technique, liner and volumetric measurements can be obtained using CBCT radiography [14].

The accuracy of other studies that have used CBCT radiography of the mandible and discriminant function analysis to estimate the sex ranges between 67% and 84.1% [36,37,38,39]. Despite the use of discriminant function analysis by many researchers in the field of forensic dentistry in predicting the sex, it has many limitations. For instance, the morphometric measurements of bone are not linear [41]. Moreover, all variables should be parametric, statistically independent, randomly sampled, and have equal sample sizes for both groups [42]. To overcome the limitations of discriminant function analysis, different ML algorithms have been used in this study, where the sample was split, with 80% dedicated to training, and the remaining 20% used to test the models.

Gabriela et al. [40], in their study, measured the ramus height and maximum length, coronoid height, gonial angle, and bigonial distance of 103 mandibular bones, using a digital caliper. The data were analyzed through an LR model that had been developed using 83 samples, and the remaining 20 samples were used for testing. The accuracy of this model in predicting the sex was 90%. This result is close to the finding of the present study. Abualhija et al. [43] used LR to estimate sex from the measurements of the ramus of the mandible from OPG radiographs. However, they predicted sex correctly with a total accuracy of 77.6%. Their result contradicted the finding of this study. This difference could be due to different radiographical techniques, and the measured parameters.

One of the most contemporary techniques used in forensics is artificial intelligence. Patil et al. [44] studied seven liner parameters in OPG radiographs of the mandible to determine the sex using three different methods (discriminant analysis, LR, and artificial neural network analysis). The result indicated that the accuracy of discriminant analysis was 69.1%, while the accuracy of LR was 69.9%, and the accuracy of the artificial neural network was 75%. The differences in their results compared to the results of the present study could be attributed to the measuring parameters, and the use of different algorithms in the current study to identify the sex.

Using ML algorithms to analyze volumetric and liner data obtained from CBCT radiographs of various structures of the maxillofacial region is a new technique to estimate the sex. Hamad et al. [45] reported that the sex could be predicted with high accuracy, reaching up to 98%, by studying the maxillary sinus morphometry with the aid of ML models. This result suggests that using ML models and CBCT radiographs is a promising approach to determining the sex.

Few studies have been conducted evaluating 3D radiography of the cranium and mandible using ML models. Toy et al. [46] evaluated twenty-five liner morphometric measurements obtained from computed tomography (CT) scans of 150 male and 150 female individual skulls and mandibles, using DT, RF, LR, linear discriminant analysis, quadratic discriminant analysis, and extra tree classifier ML algorithms. The results indicated that the sex could be predicted successfully, with a high accuracy (90%). The result of the study conducted by Toy et al. is similar to the sex prediction accuracy of the present research.

Regarding the reliability of this study, intra-examiner calibration was used, to assess the examiner’s reproducibility, and a tenfold cross-validation test was applied, to evaluate the performance of the ML models. The small sample size, and the fact that the sample belonged to one ethnic group, are considered the main limitations of this research.

## 5. Conclusions

The identification of deceased persons is essential for social and legal closure. Hence, sex determination is a crucial step in human identification, as it narrows the search for identity by excluding nearly one-half of the cases. Accordingly, much research has been conducted to find an accurate, fast, and cost-effective technique to estimate the sex. The findings of this study indicate that the RCorGonLD, and mean CorGonLD are the most reliable parameters for predicting the sex with high accuracy. Interestingly, the sex can be quickly determined with high accuracy, up to 90%, using the ML algorithm GNB. However, further studies with larger sample sizes and racial diversity are needed, to support the result of this study.

## Figures and Tables

**Table 1:** The mean ± SD of the tested parametric measurements of the mandible, concerning both sexes.

Parameters	Sex	Mean ± SD	*p* Value
BiconB (mm)	Female	79.488 ± 4.242	0.001
	Male	83.889 ± 5.549	
BigonB (mm)	Female	89.204 ± 5.021	0.001
	Male	96.411 ± 6.64	
BicorB (mm)	Female	90.787 ± 4.991	0.001
	Male	97.098 ± 5.521	
RCorGonLD (mm)	Female	56.935 ± 4.025	0.001
	Male	65.557 ± 5.008	
LCorGonLD (mm)	Female	56.905 ± 4.321	0.001
	Male	65.431 ± 5.068	
Mean CorGonLD (mm)	Female	56.92 ± 4.042	0.001
	Male	65.494 ± 4.885	
LGonMenLD (mm)	Female	80.971 ± 4.131	0.001
	Male	85.665 ± 5.277	
Mean GonMenLD (mm)	Female	81.394 ± 3.884	0.001
	Male	86.088 ± 5.012	

**Table 2:** The median ± IQR of the tested non-parametric measurements of the mandible, concerning both sexes.

Parameters	Sex	Median ± IQR	*p* Value
Vol (mm^3^)	Female	46,694.9 ± 9621.6	0.001
	Male	54,810.5 ± 13,564.1	
SA (mm^2^)	Female	16,868.3 ± 2222.1	0.001
	Male	19,522.5 ± 2701.3	
SA/Vol	Female	0.363 ± 0.05	0.044
	Male	0.353 ± 0.05	
RGonMenLGonA (mm)	Female	66.75 ± 5.58	0.008
	Male	68.7 ± 6.7	
RGonMenLD (mm)	Female	81.818 ± 4.071	0.001
	Male	86.512 ± 5.107	

**Table 3:** The AUC, cut-off values, *p* value, sensitivity, and specificity of all parameters.

Parameters	AUC (%95 CI)	Cut-Off	*p* Value	Sensitivity	Specificity
Vol	0.774 (0.711–0.836)	50,097.6	0.001	0.683	0.692
SA	0.888 (0.845–0.931)	18,272.45	0.001	0.769	0.788
SA/Vol	0.419 (0.341–0.497)	0.3587	0.044	0.442	0.452
BiconB	0.731 (0.664–0.799)	81.395	0.001	0.663	0.654
BigonB	0.817 (0.756–0.878)	93.01	0.001	0.769	0.779
BicorB	0.798 (0.739–0.858)	93.92	0.001	0.740	0.740
RGonMenLGonA	0.607 (0.529–0.684)	67.85	0.008	0.615	0.606
RCorGonLD	0.914 (0.874–0.954)	61	0.001	0.846	0.865
LCorGonLD	0.901 (0.86–0.943)	60.775	0.001	0.798	0.837
Mean CorGonLD	0.913 (0.873–0.952)	60.6025	0.001	0.837	0.817
RGonMenLD	0.761 (0.696–0.826)	83.29	0.001	0.692	0.683
LGonMenLD	0.763 (0.698–0.828)	83.075	0.001	0.712	0.702
Mean GonMenLD	0.772 (0.709–0.836)	83.2925	0.001	0.712	0.702

**Table 4:** The precision, recall, f1-score, training accuracy, and testing accuracy of the ML algorithms.

ML Algorithm	Sex	Precision	Recall	F1-Score	Training Accuracy	Testing Accuracy
LR	Female	0.86	0.90	0.88	0.89	0.88
	Male	0.90	0.86	0.88		
KNN	Female	0.76	0.95	0.84	0.87	0.83
	Male	0.94	0.73	0.82		
GNB	Female	0.86	0.95	0.90	0.84	0.90
	Male	0.95	0.86	0.90		
DT	Female	0.77	0.85	0.81	1	0.80
	Male	0.85	0.77	0.81		
RF	Female	0.83	0.95	0.88	0.99	0.88
	Male	0.95	0.82	0.88		

**Table 5:** The tenfold cross-validation of the testing accuracy of all the ML algorithms.

Testing Set	GNB	RF	DT	LR	KNN
1	0.881	0.857	0.810	0.881	0.857
2	0.857	0.833	0.881	0.929	0.881
3	0.762	0.810	0.833	0.857	0.810
4	0.810	0.833	0.786	0.833	0.857
5	0.810	0.810	0.786	0.738	0.833
6	0.833	0.786	0.786	0.738	0.762
7	0.905	0.905	0.881	0.905	0.929
8	0.857	0.857	0.762	0.905	0.857
9	0.786	0.786	0.786	0.810	0.762
10	0.905	0.929	0.833	0.881	0.929
Mean ± SD	0.84 ± 0.049	0.84 ± 0.048	0.814 ± 0.042	0.848 ± 0.068	0.848 ± 0.059

## Data Availability

The datasets used and analyzed during the current study are available from the corresponding author upon reasonable request.
